# CREATING A UNIFIED NATIONAL STRUCTURE FOR SAVVY’S DISSEMINATION AND SUSTAINABILITY

**DOI:** 10.1093/geroni/igad104.1019

**Published:** 2023-12-21

**Authors:** John Hobday, Carey Wexler Sherman

**Affiliations:** Savvy Systems, LLC, Minneapolis, Minnesota, United States; Savvy Systems, LLC, Minneapolis, Minnesota, United States

## Abstract

Intervention efforts, including established evidence-based programs (EBP), often can’t support or monitor programmatic fidelity or sustainability when external funding ends. Delivery can drift substantially or cease altogether. This paper describes the development of an authorized entity dedicated to sustain and scale Savvy Caregiver® with fidelity that was highly informed by efforts described in previous papers. The initial stage involved collaborating with the Offices of Technology Commercialization at University of Minnesota (primary copyright holder) and Emory University (lead adaptation institution). Inter-Institutional Agreements (IIA) outlined guidelines for copyright and trademark compliance, research, fidelity management, and content updates. Next, Savvy Systems, LLC, was launched to document current availability and reach of Savvy and manage the training and certification of Savvy interventionists and distribution curricula and materials through a single portal (www.savvycaregiver.com). Fidelity and essential program delivery skills are now assessed and supported via a new Savvy Fidelity Certification Exam and fidelity webinars. Finally, a flexible licensing plan for organizations and researchers ensures legal use of and access to updated authorized Savvy materials. Creation of the first national database of Savvy sites, featured on the website and the Best Practices Caregiving EBP portal (www.benrose.org), enables direct referrals and other support for Savvy sites and caregivers. Taken as a whole, this type of structure is expected to play an instrumental role in supporting the long-term fidelity, viability, and expansion of Savvy Caregiver. Adaptation and application of such processes may inform other translational and EBP efforts to ensure programmatic integrity and sustained use over time.

